# Development and external validation of nomograms for predicting individual survival in patients with ovarian clear cell carcinoma

**DOI:** 10.1002/cam4.5853

**Published:** 2023-03-27

**Authors:** Xiaoshi Liu, Huaiwu Lu, Ying Zhou, Xiaoran Long, Qing Li, Guanglei Zhuang, Xia Yin, Wen Di

**Affiliations:** ^1^ Department of Obstetrics and Gynecology, Ren Ji Hospital, School of Medicine Shanghai Jiao Tong University Shanghai China; ^2^ Shanghai Key Laboratory of Gynecologic Oncology, Ren Ji Hospital, School of Medicine Shanghai Jiao Tong University Shanghai China; ^3^ Department of Gynecological Oncology, the Affiliated Cancer Hospital, School of Medicine University of Electronic Science and Technology of China, Sichuan Cancer Hospital & Institute Chengdu China; ^4^ Department of Gynecologic Oncology, Sun Yat‐Sen Memorial Hospital Sun Yat‐Sen University Guangzhou China; ^5^ Department of Obstetrics and Gynecology, Division of Life Sciences and Medicine, The First Affiliated Hospital of USTC University of Science and Technology of China Hefei China; ^6^ State Key Laboratory of Oncogene and Related Genes, Shanghai Cancer Institute, Ren Ji Hospital, School of Medicine Shanghai Jiao Tong University Shanghai China

**Keywords:** nomogram, ovarian clear cell carcinoma, overall survival, prognostic factor, progression‐free survival

## Abstract

**Purpose:**

Ovarian clear cell carcinoma (OCCC) is a distinct and highly malignant subtype of ovarian cancer with high individual heterogeneity in survival that requires specific prognostic predictive tools. Thus, this study aimed to construct and validate nomograms for predicting individual survival in OCCC patients.

**Methods:**

In total, 91 patients with OCCC who were diagnosed and treated at Renji Hospital between 2010 and 2020 were extracted as the training cohort, then 86 patients from the First Affiliated Hospital of USTC were used as the external validation cohort. Prognostic factors that affect survival were identified using least absolute shrinkage and selection operator regression. Nomograms of progression‐free survival (PFS) and overall survival (OS) were then established with the Cox regression model and the performance was subsequently evaluated using the concordance index (C‐index), calibration plots, decision curve analysis (DCA), and risk subgroup classification.

**Results:**

Advanced tumor, ascites of >400 mL, lymph node‐positive, CA199 of >142.3 IU/mL, and fibrinogen of >5.36 g/L were identified as risk factors for OS while advanced tumor, ascites of >400 mL, lymph node‐positive, and fibrinogen of >5.36 g/L were risk factors for PFS. The C‐indexes for the OS and PFS nomograms were 0.899 and 0.731 in the training cohort and 0.804 and 0.787 in the validation cohort, respectively. The calibration plots showed that nomograms could provide better consistency in predicting patient survival than the FIGO staging system. DCA also demonstrated that nomograms were more clinically beneficial than the FIGO staging system. Additionally, patients could be classified into two risk groups based on scores using nomograms, with significant survival differences.

**Conclusions:**

We developed nomograms that could more objectively and reliably predict the individual survival of patients with OCCC compared with the FIGO staging system. These tools might assist in clinical decision‐making and management of patients with OCCC to improve their survival outcomes.

## Graphical abstract

The nomograms provide a possible way to estimate the survival of OCCC patients and can identify early‐stage patients with high‐risk who may require adjuvant chemotherapy. In future, the nomograms are expected to help gynecological oncologists formulate more individualized strategies for adjuvant treatment and follow‐up schedules to improve the clinical prognosis of patients with OCCC.

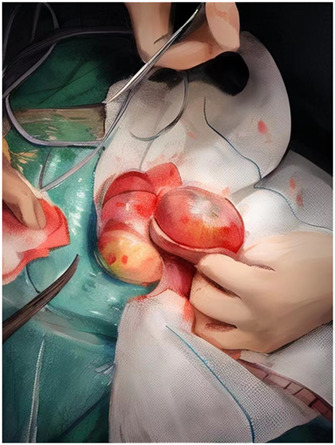

## 1 INTRODUCTION

Ovarian clear cell carcinoma (OCCC) is a relatively rare and distinct histological type of EOC with unique genetic characteristics compared to high‐grade serous carcinoma (HGSC).^1^, ^2^ It has a higher prevalence of up to 11%–30% in East Asia, while OCCC only accounts for 5%–10% of EOC in North America, which may be due to the higher endometriosis incidence in the Asian population.^3^, ^4^, ^5^ Despite a lower response to platinum‐based chemotherapy and a high heterogeneity in survival outcomes, OCCC is still treated and monitored in the same way as HGSC.^6^, ^7^, ^8^ For this reason, the value of adjuvant chemotherapy same as HGSC is still being debatable, particularly for patients with stage IA disease: most of patients tend to have a favorable prognosis while it is still regarded as a high‐grade tumor with a high risk of recurrence.^9^, ^10^, ^11^ So more information about this rare type is needed to better understand the prognosis for OCCC patients and improve the clinical evaluation and management of OCCC. However, due to the relatively low incidence, there is no specific prediction model to assess the prognosis of OCCC patients.

The International Federation of Gynecology and Obstetrics (FIGO) staging system is most frequently used to evaluate the prognosis of OCCC patients. However, there is significant heterogeneity among OCCC patients in terms of clinicopathological information, such as the presence of endometriosis, venous thromboembolism, or residual disease postoperatively, which makes FIGO staging a poor predictor of individual survival risk.^12^, ^13^, ^14^ Additionally, the conventional biomarker CA125 levels are frequently mildly elevated in patients with EOC, which is a poor OCCC biomarker and predictor.^15^ Therefore, a personalized model is required for patients with OCCC to improve disease risk subclassification and prognosis prediction.

The nomogram, a convenient tool to generate a probability of a clinical event based on specific characteristics of a patient and his or her disease has been widely used for cancer prognosis due to its value in risk stratification and personalized treatment. To our knowledge, a validated nomogram for patients with OCCC is unavailable. Hence, this study aimed to construct and validate novel nomograms for OCCC to predict their individual survival and distinguish low‐ and high‐risk patients.

## 2 MATERIALS AND METHODS

### 2.1 Patients

**FIGURE 1:**
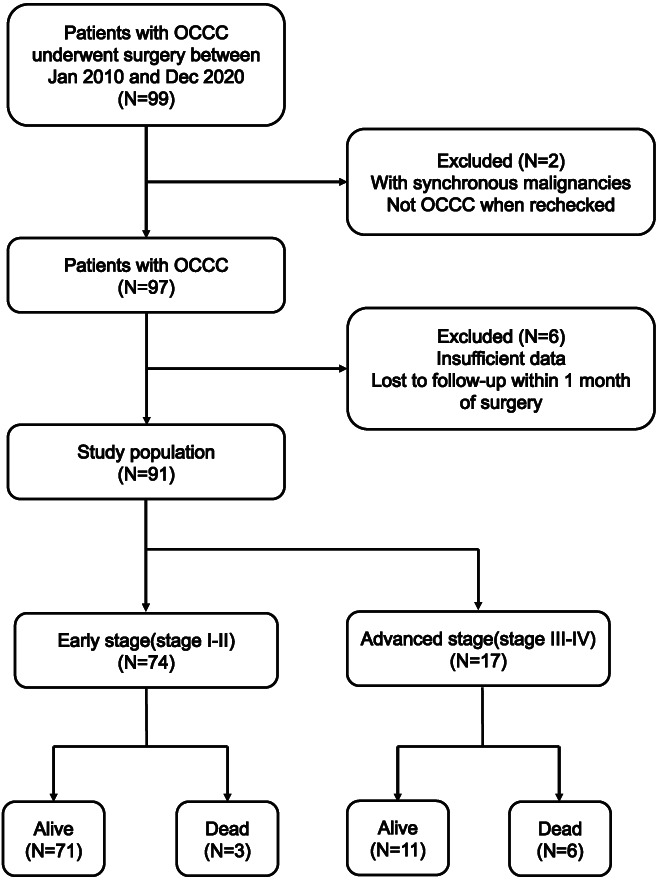
The inclusion process and clinical outcomes of the 91 patients evaluated in this study. Survival data were last updated on July 1, 2021.

This study retrospectively reviewed the data from the Hospital Information System to identify all the patients who received initial treatment with a pathological diagnosis of OCCC at Renji Hospital as the training cohort (Figure 1). Concurrently, 86 patients with OCCC from the First Affiliated Hospital of USTC were extracted as an external validation cohort. Patients aged ≥18 years and diagnosed with OCCC from January 2010 to December 2020 that is pathologically confirmed by two gynecologic pathologists were included. The exclusion criteria included (1) patients who had insufficient data; (2) with synchronous malignancies; (3) received neoadjuvant chemotherapy; and (4) lost to follow‐up within 1 month of surgery. All patients were restaged according to the International Federation of Gynecology and Obstetrics (FIGO) 2014 staging system. The medical records of all patients were reviewed. Collected data included demographic information, clinical preoperative laboratory tests, treatment (surgical procedure, chemotherapy), and dates and status of follow‐up (recurrence or death). Survival data were censored on July 1, 2021.

### 2.2 Statistical analysis

The least absolute shrinkage and selection operator (LASSO) method was applied to identify prognostic factors for OS and PFS to avoid over‐fitting. By decreasing some regression coefficients to zero, the LASSO method regularizes model parameters. Following the shrinkage, comes the factor selection phase, during which all non‐zero values are chosen to be incorporated in the model. Since the method involves coefficient shrinking, which lowers variance and minimizes bias, accuracy increases. The OS‐predicting nomogram and PFS‐predicting nomogram were visually presented based on the beta value of these factors. The nomogram performance was externally validated in the validation cohort. The discrimination strength was assessed by the concordance index (C‐index). Calibration curves were generated to evaluate the consistency between the predicted and actual survival probabilities with 1000 bootstrap resamples. Additionally, decision curve analyses (DCAs) were utilized to examine the nomograms' clinical usefulness compared to the FIGO staging. Moreover, OS and PFS risk classification systems were established corresponding to nomograms in the training cohort. The Kaplan–Meier curves tests were used to compare the survival in patients with different risk classification. All statistical analyses were performed with R version 3.6.3 and a *p*‐value of <0.05 was considered statistically significant.

## 3 RESULTS

### 3.1 Patient characteristics and survival outcomes

In total, 91 patients who were diagnosed with OCCC from January 2010 to December 2020 were enrolled as the training cohort. The clinicopathological characteristics and preoperative laboratory tests are presented in Tables 1 and 2. Expectedly, the early‐stage disease made up the majority of the population, wherein 74 (81.4%) patients had Stage I or II disease. There were nine (9.9%) patients with endometriosis‐associated OCCC according to the Scott criteria. The median CA‐125 level was 58.0 IU/mL (range, 5.81–2020.0 IU/mL) in all patients, which was 52.0, 60.9, 102.0, and 611 IU/mL in Stages I–IV, respectively. Elevated CA199 (>37 IU/mL) was observed in 25 patients, with a median concentration of 15.4 IU/mL (range, 0.5–1060.0 IU/mL). The patients' blood fibrinogen levels range from 1.60 to 17.10 mg/dL, with a median level of 3.65 mg/dL. A total of 86 patients from another center were served to validate nomograms, and the characteristics and preoperative laboratory tests of these patients are shown in Table 3.

**TABLE 1:** Baseline demographics of patients (*n* = 91).

Characteristics	All (%)
All cases	91
Age at diagnosis, median (range, years)	56 (32–79)
Menopausal state	
Y	67 (73.6%)
N	24 (26.4%)
FIGO Stage	
I	60 (66.0%)
II	14 (15.4%)
III	15 (16.5%)
IV	2 (2.1%)
Endometriosis	9 (9.9%)
Thrombosis	10 (11.0%)
Ascites	
Y	37 (40.6%)
>400 mL	16 (43.2%)
≤400 mL	21 (56.8%)
N	54 (59.3%)
Chronic disease comorbidities	
Hypertension	21 (23.1%)
Diabetes	7 (7.7%)
Heart disease	4 (4.4%)

**TABLE 2:** Preoperative laboratory tests of OCCC patients.

	Overall	FIGO I	FIGO II	FIGO III	FIGO IV
	(*N* = 91)	(*N* = 60)	(*N* = 14)	(*N* = 15)	(*N* = 2)
CA125 (U/mL)					
Normal < 35					
Median [Min, Max]	58.0 [5.8, 2020.0]	52.0 [5.8, 603.0]	60.9 [26.5, 364.0]	102.0 [32.8, 2020.0]	611.0 [413.0, 809.0]
CA199 (U/mL)					
Normal < 37					
Median [Min, Max]	15.4 [0.5, 1060.0]	16.6 [0.5, 728.0]	9.41 [0.9, 1060.0]	12.0 [1.9, 246.0]	133.0 [4.3, 262.0]
CEA (ng/mL)					
Normal <5					
Median [Min, Max]	1.33 [0.20, 6.30]	1.36 [0.20, 5.85]	1.20 [0.21, 5.59]	1.35 [0.66, 6.30]	1.07 [0.62, 1.52]
AFP(ng/mL)					
Normal < 25					
Median [Min, Max]	2.99 [0.70, 12.6]	3.15 [0.70, 12.6]	3.46 [1.67, 12.10]	2.67 [0.98, 12.10]	1.85 [0.91, 2.79]
Albumin(g/L)					
Normal 35–50					
Median [Min, Max]	42.0 [27.5, 69.1]	42.5 [27.5, 52.1]	42.6 [37.2, 46.0]	38.8 [29.5, 69.1]	31.2 [27.9, 34.4]
Platelet (10^^^9/L)					
Normal 100–300					
Median [Min, Max]	255 [117, 539]	255 [134, 505]	248 [117, 539]	315 [181, 376]	446 [424, 467]
D‐dimer (mg/L)					
Normal <0.5					
Median [Min, Max]	0.53 [0.01, 6.03]	0.51 [0.01, 6.03]	0.48 [0.08, 3.57]	0.54 [0.10, 4.38]	1.95 [1.93, 1.97]
Blood fibrinogen (mg/dL)					
Normal 2–4					
Median [Min, Max]	3.65 [1.60, 17.10]	3.42 [1.60, 17.10]	4.68 [2.06, 7.93]	4.66 [2.08, 6.88]	6.71 [6.18, 7.24]

**TABLE 3:** Clinical characteristics of the external cohort (*n* = 86).

	Cases	%
All cases	86	
Age at diagnosis^a^	50 [25, 70]	
FIGO staging		
I	56	65.1
IA	25	29.1
IB	2	2.3
IC	29	33.7
II	3	3.5
IIA	0	0.0
IIB	3	3.5
III	24	27.9
IIIA	4	4.7
IIIB	1	1.1
IIIC	19	22.1
IV	3	3.5
Ascites		
Y	39	45.3
N	47	54.7
Lymph nodes positive	3	3.5
Endometriosis	16	18.6
Thrombosis	8	9.3
Chronic disease comorbidities		
Hypertension	3	3.5
Diabetes	16	18.6
Preoperative laboratory tests		
CA125 (IU/mL)^a^	105.0 [9.2, 9035.0]	
CA199 (IU/mL)^a^	31.0 [0.6, 96649.0]	
Albumin (g/L)^a^	40.8 [31.1, 51.6]	
Platelet (109/L)^a^	249 [73, 731]	
Fibrinogen (g/L)^a^	3.30 [1.77, 9.66]	

^a^ Median, range.

### 3.2 Identification of prognostic factors and nomograms construction

**FIGURE 2:**
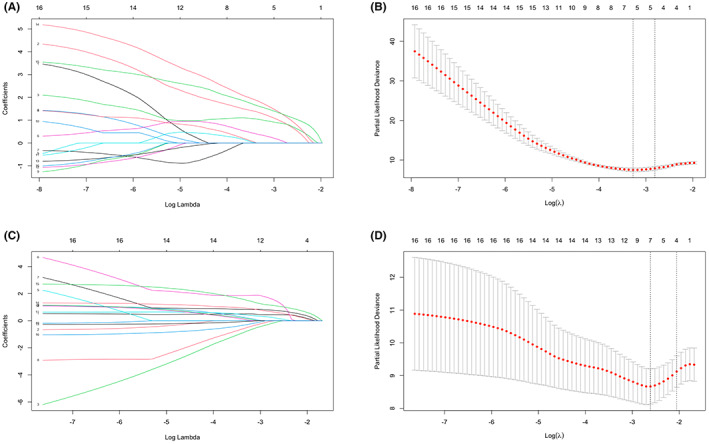
LASSO regression identified prognosis‐related factors in OCCC patients. (A) and (B) LASSO Cox regression was performed to identify the factors closely associated with the OS of OCCC. (C) and (D) LASSO Cox regression was performed to identify the factors closely associated with the PFS of OCCC.

**FIGURE 3:**
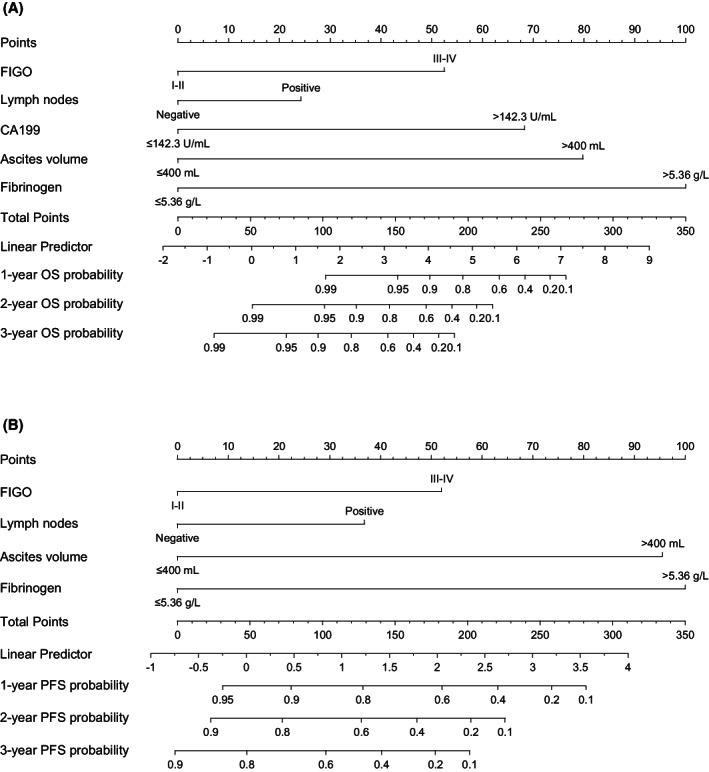
Nomograms for predicting 1‐, 2‐, and 3‐year (A) OS and (B) PFS of OCCC patients.

A total of 16 variables that may affect the prognosis of patients with OCCC were included in the LASSO regression. Advanced tumor, lymph node‐positive, ascites (>400 mL), CA199 (>146.3 IU/mL), and fibrinogen (>5.36 g/L) were identified for OS prognostic factors (Figure 2A,B). Similarly, four factors, including advanced tumor, lymph node positivity, ascites (>400 mL), and fibrinogen (>5.36 g/L) were identified as PFS prognostic factors (Figure 2C,D). Based on the above, the predictive model by the multivariate COX regression was virtually presented in the form of nomograms to predict 1‐ to 3‐year OS and PFS (Figure 3).

### 3.3 Nomogram validation and risk classification system

**FIGURE 4:**
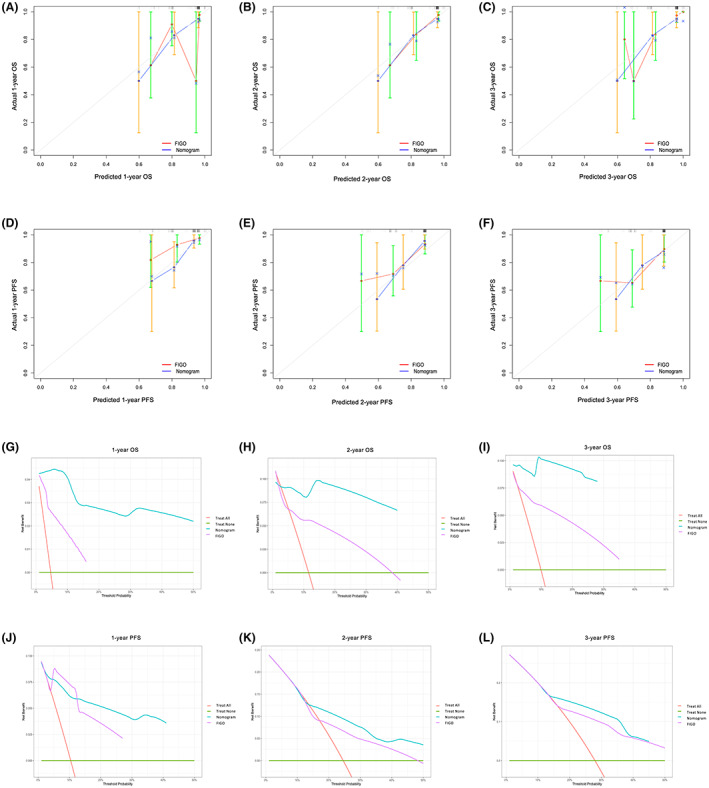
External validation of nomograms. (A–C). Calibration plots of the nomogram for 1‐, 2‐, and 3‐year OS in the in the validation cohort. (D–F). Calibration plots of the nomogram for 1‐, 2‐, and 3‐year PFS in the in the validation cohort. (G–I). DCA curves determined that the OS nomogram can provide optimal clinical decision‐making benefits than the FIGO staging. (J–L). DCA curves determined that the PFS nomogram can provide optimal clinical decision‐making benefits than the FIGO staging.

The OS and PFS C‐indexes were 0.899 (95% CI, 0.850–0.948) and 0.731 (95% CI, 0.625–0.837) in the training cohort while 0.804 (95% CI, 0.642–0.967) and 0.787 (95% CI, 0.637–0.938) in the validation cohort, respectively. The calibration curves at 1–3 years showed that both OS and PFS nomogram had a better predictive ability to predict the survival probabilities of patients with OCCC than the FIGO staging in the validation cohort (Figure 4A–F). DCA curves confirmed that the nomogram models can result in more favorable potential clinical benefits compared with the FIGO staging system at 1‐ to 3‐year time points (Figure 4G–L).

**FIGURE 5:**
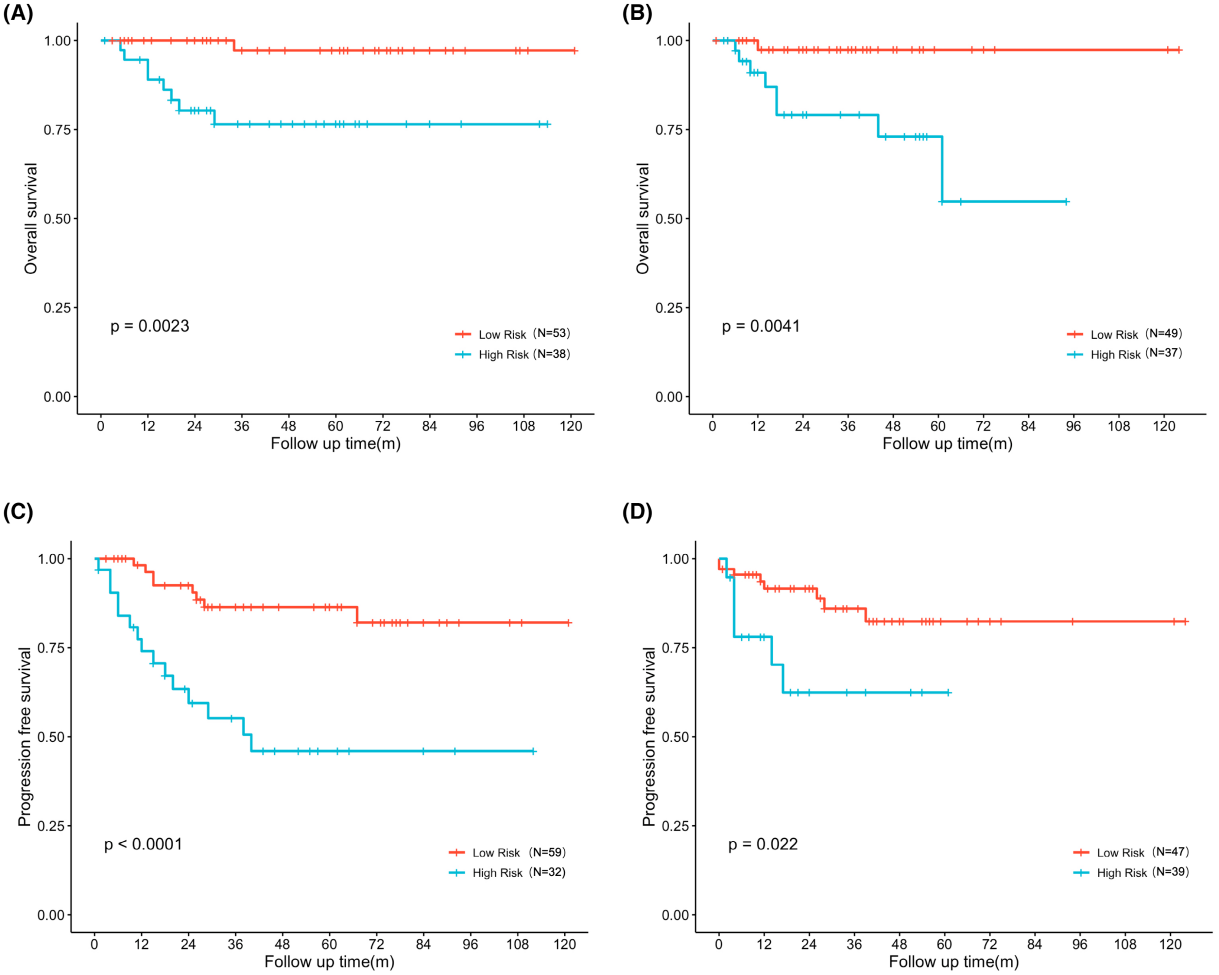
Kaplan–Meier curves of OS and PFS for risk stratification. Kaplan–Meier curves of OS for risk stratification in the training cohort (A) and the external validation cohort (B). Kaplan–Meier curves of PFS for risk stratification in the training cohort (C) and the external validation cohort (D).

Additionally, the risk classification system for OS and PFS was also developed based on nomograms to divide patients into two distinct prognostic groups. There were significant survival differences between the low‐ and high‐risk groups for OS and PFS in the training cohort as well as validation cohort, implying the nomogram's outstanding ability to identify the sickest patients with OCCC (Figure 5).

## 4 DISCUSSION

OCCC is a relatively uncommon type of EOC with distinctive biological and clinical features.^3^, ^4^, ^5^ Accurate assessment of patients' prognosis is essential for selecting appropriate therapy and planning follow‐up care. The FIGO staging system is a key prognostic determinant in OCCC in routine clinical practice. However, high heterogeneity in survival outcomes even among patients with the same stages suggests that the present FIGO staging system is inadequate for prognosis. Hence, the present study constructed nomograms to assess individual survival for patients with OCCC and reported several notable findings. More excellent nomogram performances than the FIGO staging were shown by calibration and DCA curves. Additionally, the nomograms enabled oncologists to identify potential patients with OCCC at increased risk.

To date, few reliable factors that predict OCCC prognosis have been identified. Our study used the LASSO regression, which could exceedingly improve its predictive accuracy, to identify prognosis‐related variables. CA125 is the most common serum biomarker for EOC, which can evaluate stage, prognosis, tumor recurrence, and treatment response. However, in the present study, 21 (23.1%) patients had a normal CA125 level and its value did not correlate with OS or PFS. In recent years, related studies have revealed elevated serum CA199 in patients with ovarian cancer,^16^, ^17^ which is an independent poor OS prognostic factor for OCCC.^18^ The present study revealed elevated CA199 (>142.3 U/mL) as a significant predictor of poor OS in patients with OCCC. More than one‐third of ovarian cancer patients developed ascites at initial diagnosis and ascites is likely to further cause chemoresistance, widespread metastasis, and decreased surgical resectability.^19^, ^20^, ^21^, ^22^ A recent study showed that HGSC patients with ascites <200 mL have lower serum CA125 levels, more optimal cytoreduction rates, and longer disease‐free intervals than those with large ascites.^23^ However, most ascites research focuses on HGSC, and determining the importance of ascites in the progression of rare EOC subtypes, such as OCCC, is currently difficult. The present study revealed that survival analysis showed ascites (>400 mL) as an independent OS‐ and PFS‐related factors for patients with OCCC. The discrepancy in the cutoff value may be attributed to our large proportion of patients with early‐stage OCCC. Each study has set its threshold based on a respective small sample size which could not be directly applied to clinical practice; thus, further studies are needed to confirm the ascites volume as a prognostic marker.

It is wildly accepted that OCCC patients are at higher risk for cancer‐associated venous thromboembolic events compared to other ovarian cancers.^13^, ^24^ Fibrinogen, as an important coagulation factor, has demonstrated its role in tumorigenesis, angiogenesis, and tumor metastasis.^25^, ^26^ Hyperfibrinogen can likely stimulate tumor angiogenesis by an inflammatory and cause a hypercoagulable state in cancer patients.^25^ The present study identified blood fibrinogen (>5.36 mg/dL) as an independent factor for PFS and OS based on our clinical data, which have not been widely addressed in previous studies. These data indicate that elevated fibrinogen plays an important role in the tumor aggressiveness and prognosis of patients with OCCC.

The FIGO staging for ovarian cancer was first published in 1973 and revised in 2014.^27^ However, ovarian cancer is not a unitary disease, but rather several different malignancies with different clinical and pathological characteristics that share a common anatomical site upon presentation. A previous study analyzed the clinical significance of the FIGO 2014 staging by including 9747 patients with EOC from 2004 to 2008.^28^ The subgroup analysis suggested a significant difference in the prognosis of patients with OCCC with stage IA versus IC1 and no difference in patients with HGSC, suggesting that IC subclassification may be too detailed. Complex staging or other excluded clinicopathological variables in staging sometimes influence the prognosis of patients with OCCC. Therefore, nomograms have been widely used in cancer prognosis prediction, which transforms complex regression equations into simple and easy‐to‐understand visual graphs, making the prediction model results more readable to facilitate patient evaluation.^29^ Based on SEER database, Chen and his team constructed nomograms to predict OS and CSS in OCCC patients.^30^ However, the main limitations using the SEER data are as follows: (1) detailed information such as preoperative tests levels, as well as chemotherapy regimens and cycles are unavailable; (2) since SEER is a US‐population based public database, the generalizability and transferability of its research results in other population may be limited; (3) the nomograms only received internal validation. This study externally validated the nomograms used to predict OS and PFS in patients with OCCC, which all revealed better predictive performance than the FIGO staging. Additionally, the scoring systems were useful to provide additional information for patient stratification and treatment strategies. Particularly, the nomograms could be used to select high‐risk patients with stage IA‐IC1 OCCC who require adjuvant chemotherapy.

To our knowledge, no other externally validated nomograms that estimated the patient survival in OCCC has been built. Moreover, the variables used to construct the nomograms are common clinicopathological (ascites volume, positive lymph node metastasis, etc.) and routine laboratory (Fibrinogen, CA199, etc.) data, which are inexpensive and readily available even in underdeveloped areas. However, our study has several limitations. This retrospective study had an inherent bias, as all patients were from China and the distribution of clinical characteristics might be different in other areas. Besides, due to some patients' lack of complete medical information and follow‐ups, our sample size was insufficient. Therefore, further external validation in other populations and prospective studies in larger cohorts are needed to verify our conclusion. Additionally, we did not include different maintenance therapy regimens such as angiogenesis inhibitors, PARP inhibitors or immune checkpoint inhibitors in the study which may have led to potential heterogeneity between patients. Last but not least, a median follow‐up time of 55 months may not extensive for a disease that predominantly presents at an early stage; thus, the event rate for OS data is relatively low. We will follow‐up with patients and update future data to further validate our findings.

In conclusion, we developed and externally validated nomograms, which effectively predict recurrence and survival for patients with OCCC, and added prognostic value to the FIGO staging. The nomograms are expected to help gynecological oncologists formulate more individualized strategies for adjuvant treatment and follow‐up schedules to improve the clinical prognosis of patients with OCCC.

## AUTHOR CONTRIBUTIONS

**Xiaoshi Liu:** Conceptualization (equal); data curation (lead); formal analysis (equal); software (lead); validation (lead); visualization (lead); writing – original draft (lead); writing – review and editing (equal). **Huaiwu Lu:** Conceptualization (equal); formal analysis (equal); validation (equal); visualization (equal); writing – original draft (equal); writing – review and editing (equal). **Ying Zhou:** Conceptualization (equal); data curation (equal); methodology (equal); validation (equal); visualization (equal); writing – original draft (equal); writing – review and editing (equal). **Xiaoran Long:** Data curation (supporting); validation (supporting); visualization (supporting); writing – review and editing (supporting). **Qing Li:** Data curation (supporting); formal analysis (supporting); methodology (supporting); validation (supporting); writing – review and editing (supporting). **Guanglei Zhuang:** Conceptualization (supporting); methodology (supporting); resources (supporting); writing – original draft (supporting); writing – review and editing (supporting). **Xia Yin:** Conceptualization (equal); funding acquisition (lead); investigation (lead); supervision (equal); writing – original draft (equal); writing – review and editing (equal). **Wen Di:** Conceptualization (lead); funding acquisition (lead); methodology (lead); project administration (lead); supervision (lead); writing – original draft (equal); writing – review and editing (equal).

## FUNDING INFORMATION

The study was funded by grants from the National Natural Science Foundation of China (NSFC) (No. 82173077 to Xia Yin, No. 81974454 to Wen Di and No. 81772770 to Wen Di), from the Shanghai Natural Science Foundation (No. 20ZR1433100 to Xia Yin), from the Shanghai Shenkang Hospital Development Center (No. SHDC2020CR3057B to Xia Yin), from the Shanghai Collaborative Innovation Center for Translational Medicine (No. TM202004 Xia Yin), from the Beijing Kuanghua foundation for the development of Chinese and Western Medicine (No. KH‐2021‐LLZX‐018 to Xia Yin), from the Shanghai Municipal Commission of Health and Family Planning (to Wen Di), from the Shanghai Municipal Key Clinical Specialty (to Wen Di).

## CONFLICT OF INTEREST STATEMENT

The authors have no conflicts of interest to declare.

## ETHICS STATEMENT

The study was approved by the Ethics Committee of Ren Ji Hospital, School of Medicine, Shanghai Jiao Tong University Shanghai (KY2021‐031) and the Ethics Committee of the First Affiliated Hospital of USTC (202203211614000188310).

## Data Availability

The datasets used or analyzed during the current study are available from the corresponding author on reasonable request.
